# Phantom Phone Signals in youths: Prevalence, correlates and relation to psychopathology

**DOI:** 10.1371/journal.pone.0210095

**Published:** 2019-01-04

**Authors:** Simone Pisano, Pietro Muratori, Vincenzo Paolo Senese, Chiara Gorga, Margherita Siciliano, Marco Carotenuto, Raffaella Iuliano, Carmela Bravaccio, Simona Signoriello, Antonella Gritti, Antonio Pascotto, Gennaro Catone

**Affiliations:** 1 Department of Neuroscience, AORN Santobono-Pausilipon, Naples, Italy; 2 IRCCS Stella Maris, Scientific Institute of Child Neurology and Psychiatry, Calambrone, Italy; 3 Department of Psychology, University of Campania "Luigi Vanvitelli", Naples, Italy; 4 Department of Mental and Physical Health and Preventive Medicine, University of Campania, Naples, Italy; 5 Department of Neonatology and Neonatal Pathology, Hospital ‘‘Loreto Mare”, Naples, Italy; 6 Department of Translational Medicine, Federico II University of Naples, Naples, Italy; 7 Faculty of Educational Sciences, Suor Orsola Benicasa University, Naples, Italy; Boys Town National Research Hospital, UNITED STATES

## Abstract

The term Phantom Phone Signals (PPS) refers to the perception of a mobile phone ringing, vibrating and blinking when in fact it did not. Data in youth are lacking, and controversies exist on whether PPS is related to psychopathology. In the present study, we showed data on the prevalence of PPS in a population (N = 2959) of students aged 10 to 14 years. We also explored the possible association between PPS and emotional or behavioural problems. Our results showed that PPS is a relatively common phenomenon with a prevalence rate of 58.9%, being more frequent in females. In univariate and multivariate analyses, we also found an association between the presence of PPS and emotional problems and temper tantrums, after accounting for relevant covariates. PPS is a relevant phenomenon to be considered in youth. It is common and may be a signal for emotional problems.

## Introduction

“Phantom Vibration” (PV) or “Phantom Ringing” (PR) refers to the mistaken perception of a phone vibrating or ringing, respectively, when in fact it did not; in previous literature, other terms were used, such as ‘‘ringxiety,” ‘‘vibranxiety”, ‘‘FauxCellArm” and the all-inclusive term Phantom Phone Signals (PPS) proposed by Tanis et al. [1], which includes all kinds of perceptions of phone signals. Although some differences were noted [1] among these authors, we did not find compelling evidence that justified considering PV and PR as different entities; thus, in the present paper, we will follow Tanis et al. [1] in considering PR and PV as different manifestations of the same overarching phenomenon, and throughout the manuscript, we refer to it as PPS.

Most of the studies agreed with PPS being a common phenomenon, with various prevalence estimates; in the only review present in the literature, the prevalence of PPS ranged between 27.4% and 89% [2]. It has been mostly studied in health professionals or university students, leaving a gap in the knowledge about this phenomenon in school-aged youths [1, 3, 4, 5, 6]. This is striking because mobile phone use is basically ubiquitous in children and adolescents (>90% of adolescents [7] and 69% of children [8]); also, younger users are more likely than older users to spend much time on their mobile phones and to suffer from mobile phone-related problems [9, 10]; moreover, in the first study on the phenomenon, Rothberg et al. [3] found younger people to be more prone to the experience of PV.

The association between PSS and psychological/psychopathological factors has been studied in adults and produced mixed findings. Drouin et al. [11] showed a small but significant relation between PPS and text-message dependency and a negative relation between frequency of PPS and conscientiousness. Lin et al. [4] showed an association between the frequency and the intensity of PPS and stress due to a medical internship, but not with anxiety or depression; in a subsequent study [5], they found that interns with severe PPS had higher levels of somatic depression and subjective and somatic anxiety symptoms than did the interns with subclinical PPS. Kruger and Djerf [6] found that PPS was associated with attachment anxiety, but not with general sensation seeking and attachment avoidance. Chen et al. [12] found an association between PPS and work-related burnout syndrome symptoms, but not with general measures of anxiety and depression. It may be that, aside the above mentioned factors previously identified, mental health problems (and related to that susceptibility to mild hallucinations) explain the presence of PPS in a youth population. Thus, examining the extent to which emotional and behavioural problems are related to PPS is an important contribution to a growing literature, which covers an increasingly salient aspect of youth’s everyday life.

Based on the above mentioned considerations, we conducted a survey in a school-based population of youth aged from 9 to 14 years, to fill the gap in the prevalence of the phenomenon in this age range. We then tested the hypothesis that PPS was related to emotional problems, to shed light on the existing controversies. We also explored, for the first time, the relationship of PPS with other behavioural problems.

## Materials and methods

The present data were drawn from a project whose aim is wider and encompasses several aspects of mental health and social phenomena (e.g., bullying and cyberbullying, mobile phone use, temperament and psychopathological features). It is a school-based population survey, settled in the metropolitan city of Naples, Italy. The present data were collected in a cross-sectional manner, while the longitudinal phase remains ongoing. Twelve middle schools across the area of interest (age range of students was approximately 10–14 years across three levels of instruction: 1^st^, 2^nd^ and 3^rd^ grade), six in the city of Naples and six in suburban areas, were recruited in the school year 2015/2016. Here, we present data from 2959 students (66.6% of the total students). In each school, the research team had a meeting with the classroom before the data collection to explain the research aims, procedure and motivate students to respond sincerely and accurately to obtain high-quality data. A researcher was always present during questionnaire administration to answer any queries. Subjects unable to read Italian and those with certified intellectual disabilities of any grade and type were excluded. Students with learning difficulties had the opportunity to fill in the questionnaire in the presence of a dedicated teacher. More details on the procedures and recruitment are reported elsewhere (see Catone et al., in press). For the purpose of the present study, we only included subjects who responded “yes” to the question of whether they had a smartphone. Subjects who had a smartphone made up 2859 of the 2959 students (96% of the total sample), which included 1457 males (51%) and 1402 females (49%). All parents signed a consent form after a thorough explanation of the procedures; the ethical committee of the Campania University “Luigi Vanvitelli” approved the study protocol.

### Measures

#### Smartphone use-related items

Three items were developed ad hoc to investigate smartphone use-related variables. Participants were asked the following questions:

PPS: “Has it ever happened that you hear or feel your smartphone ringing or vibrating, but do not see anything when you check your phone?”. Those who responded ‘‘yes” were then asked ‘‘How often did you feel it?” and they could answer on a Likert scale from 0 to 10. This item is slightly different from other studies, which asked a judgement on how bothersome the events felt to the respondent [4, 5, 6], because we tried to capture the intensity of the phenomenon in a continuous measure that may reflect the personal view of the experience, without conveying a negative meaning to the question.

Smartphone use: “has it ever happened that you use the smartphone for a time period that you have judged excessive?” Those who responded ‘‘yes” were then asked ‘‘How often?”, and they could answer on a Likert scale from 0 to 10. We used both (dichotomous and continuous) variables in the analyses, which yielded similar results; we only showed the model with the continuous version of the variable.

Checking the smartphone: “has it ever happened that you continuously check your smartphone in order to grasp as many conversations as possible on social media (like Facebook or Twitter)?” Those who responded ‘‘yes” were then asked ‘‘How often?” and they could answer on a Likert scale from 0 to 10. We used both dichotomous and continuous variables in the analyses, which yielded similar results; we only showed the model with the continuous version of the variable.

#### Psychopathology

We used the self-report Italian version of the Strength and Difficulties Questionnaire (SDQ) [13] to assess general psychopathology. The SDQ comprised 25 items and provided five subscales: emotional symptoms, conduct problems, hyperactivity-inattention problems, peer problems, and prosocial behaviour. Each item uses a three-point ordinal format to be answered with one of the following: 0 = not true; 1 = somewhat true; and 2 = certainly true. The mean score for each subscale was then calculated (range 0–10) and used in the analyses. The psychometric properties of the self-report version of the SDQ are generally good across studies [14, 15, 16, 17]. The alpha values for each subscale were 0.72 for the emotional symptoms subscale, 0.46 for the conduct problems subscale, 0.59 for the hyperactivity-inattention subscale, 0.55 for the peer problems subscale and 0.66 for pro-social behaviour subscale. The alpha values were generally consistent with those reported in the literature [17].

### Data analyses

Continuous variables were reported as either the mean and standard deviation (SD), as assessed by the Shapiro-Wilk test, and compared with t-tests. Categorical variables were reported as absolute numbers and percentages and compared with Pearson’s chi-squared tests. To test the factors associated with the presence/absence of PPS, we fitted a logistic regression model with the dichotomous PPS variable as the dependent variable and age, gender, smartphone-related variables (use and check) and the scores of each of the psychopathology subscales (emotional problems, conduct problems, hyperactivity, peer problems, pro-social behaviour) as independent variables. As the alpha of conduct problems subscale was poor we tried to delete items for improving it; as we could not achieve a better alpha, we included all items separately into the model. To test for the variables that may better explain the variance in the intensity of the phenomenon a multivariable regression model was also fitted with the Likert scale of the PPS as the dependent variable and the same independent variables; we tested two models, one with only subjects who reported having experienced PPS, one with the whole sample (showed in supplementary material). Models were checked for the presence of collinearity by calculating the variance inflation factor. All analyses were run in SPSS Version 20 (IBM SPSS version 20, Armonk, NY).

## Results

PPS was reported by 1685 of the students (58.9%), including 896 females (53.1%) and 789 males (46.8%) (chi squared = 28.1, p<0.000). Univariate comparisons revealed significant differences in age, gender, smartphone-related and psychopathological variables between those who reported PPS and those who did not (see Table 1).

**Table 1:** Descriptive statistics of the sample.

		PPS		
	Total (n = 2859)	Yes (n = 1685)	No (n = 1174)	p-value
Male gender, n (%)	1457 (51%)	789 (47%)	668 (57%)	<0.000
Age, mean (SD)	11.95 (0.95)	11.9 (0.9)	11.7 (0.9)	<0.000
Excessive Smartphone use, n (%)	2021 (70%)	702 (24%)	472 (16%)	<0.000
How often? mean (SD)	5 (3.1)	5.6 (3)	4.1 (3)	<0.000
Check the smartphone, n (%)	1327 (46%)	954 (33%)	373 (13%)	<0.000
How often? mean (SD)	3.2 (3.5)	3.9 (3.6)	2.1 (3)	<0.000
SDQ-EP, mean (SD)	3 (2.4)	3.3 (2.5)	2.59 (2.3)	<0.000
SDQ-CP, mean (SD)	2.2 (1.6)	2.5 (1.6)	1.9 (1.5)	<0.000
SDQ-HY, mean (SD)	3.3 (2.1)	3.6 (2.1)	2.9 (2)	<0.000
SDQ-PP, mean (SD)	1.9 (1.8)	2 (1.8)	1.7 (1.7)	<0.000
SDQ-PSB, mean (SD)	7.6 (1.9)	7.5 (1.9)	7.7 (1.9)	0.015

PPS: Phantom Phone Signal; SDQ-EP: SDQ Emotional Problems; SDQ-CP: SDQ Conduct Problems; SDQ-HY: SDQ Hyperactivity; SDQ-PP: SDQ Peer Problems; SDQ-PSB: SDQ Pro-social Behaviour.

The logistic regression model revealed that emotional problems and temper tantrums were the two psychopathological variables associated with the presence of PPS (OR = 1.06 and 1.1 respectively, p = 0.005 and p = 0.05), after controlling for age, gender and smartphone-related variables (all significant predictors). This suggested that older age, female gender, higher time spent on the smartphone and more frequent checking of the smartphone (self-reported), were all factors linked to PPS; after accounting for those factors, emotional problems and temper tantrums were still significant predictors. The model accounted for 24% of the variance. See Table 2.

**Table 2:** Logistic regression model with the dependent variable PPS (dichotomous), N = 2859.

PPS		
	OR (95% CI)	p-value
Age	1.2 (1.1–1.3)	0.000
Gender (Male/Female)	0.7 (0.6–0.9)	0.002
Smart-time	1.08 (1.0–1.1)	0.000
Smart-check	1.09 (1.0–1.1)	0.000
SDQ-EP	1.06 (1.0–1.1)	0.005
SDQ-HY	1.04 (0.9–1)	0.08
SDQ-PP	0.99 (0.9–1)	0.9
SDQ-PSB	0.99 (0.9–1)	0.9
Tantrums	1.1 (0.9–1.3)	0.05
Obeys	0.8 (0.77–1)	0.1
Fights	0.9 (0.8–1.1)	0.8
Lies	1 (0.9–1.2)	0.5
Steals	1 (0.8–1.3)	0.4
R^2^	0.24	

OR: odds ratios. PPS: Phantom Phone Signal; SDQ-EP: SDQ Emotional Problems; SDQ-CP: SDQ Conduct Problems; SDQ-HY: SDQ Hyperactivity; SDQ-PP: SDQ Peer Problems; SDQ-PSB: SDQ Pro-social Behaviour.

More specifically, the second regression model (1685 subjects), exploring factors linked to the intensity of the phenomenon (self-reported), partially confirmed previous results: gender was not a significant predictor, however, time spent on the smartphone (B = 0.21, p<0.000), checking the smartphone (B = 0.23, p<0.000), emotional problems (B = 0.13, p<0.000) and temper tantrums (B = 0.23, p = 0.02) were still significant predictors. The model accounted for 25% of the variance. See Table 3. Model including all the subjects yielded the same pattern of results (see complementary material).

**Table 3:** Multivariate regression model with the dependent variable PPS (Likert scale), N = 1685.

PPS (n. 1685)		
	B (95% CI)	p-value
Age	0.13 (0.00–0.2)	0.049
Gender (Male/Female)	0.17 (-0.8–0.4)	0.194
Smarttime	0.22 (0.17–0.26)	0.000
Smartcheck	0.23 (0.19–0.27)	0.000
SDQ-EP	0.09 (0.03–1.15)	0.003
SDQ-HY	0.01 (-0.05–0.8)	0.613
SDQ-PP	-0.01 (-0.09–0.05)	0.637
SDQ-PSB	0.05 (-0.01–1.12)	0.117
Tantrum	0.23 (0.03–0.42)	0.021
Obey	-0.10 (-0.3–0.1)	0.345
Fight	0.20 (-0.06–0.4)	0.133
Lie	-0.04 (-0.2–1.1)	0.636
Steal	0.05 (-0.2–0.3)	0.696
R^2^	0.25	

PPS: Phantom Phone Signal; SDQ-EP: SDQ Emotional Problems; SDQ-CP: SDQ Conduct Problems; SDQ-HY: SDQ Hyperactivity; SDQ-PP: SDQ Peer Problems; SDQ-PSB: SDQ Pro-social Behaviour.

## Discussion

The present study explored the phenomenon of PPS in a large and representative sample of school-aged youth. To the best of our knowledge, this is the first study investigating this phenomenon in 10- to 14-year old Italian students. Data showed that the prevalence of experiencing phantom ringing or vibrating was relatively common (58.9% of the subjects), was more frequent in females and tended to be more common with increasing age. The prevalence rate is not far from that reported in studies with older subjects (67% reported by Rothberg [3] and 78% by Lin [4]). Thus, the high frequency has also been confirmed in youth, and these data are relevant considering the widespread use of smartphones in pre-adolescence. Mohammadbeigi [18] reported a higher female prevalence for vibration and male prevalence for ringing; other studies did not find differences across genders (taking vibration and ringing together [1]). Future studies should elucidate whether the higher prevalence in females in our study is an age-related result or whether the result could be explained by our larger sample size; that is, other studies did not have enough statistical power to detect the gender difference. Epidemiological findings are highly relevant in light of the subsequent results regarding the relationship between PPS and emotional symptoms. We tried to elucidate previous controversies in the literature studying a larger sample size with a well-established measure of psychopathology. Our regression models revealed an association between the presence of PPS and both emotional problems and temper tantrums; these associations survived the control with age, gender, smartphone use and check and other psychopathology scales. Additionally, both models with dichotomous and dimensional variables showed converging results, thereby strengthening the findings. Thus, we confirmed and extended to a younger population, previous data from the literature reporting an association with PPS and affective symptoms [4, 5, 6]. Lin et al. [5] demonstrated an association between somatic anxiety and depression and severe PPS in medical interns during an internship. The authors discussed the cognitive mechanism from which auditory hallucinations may arise in non-schizophrenic subjects (top-down mechanisms, abnormalities in executive inhibition, and negative emotions [19]). We noted the relevance of the latter factor, negative emotions, which seems to provide the emotional background to the phenomenon. This is in line with recent studies on psychotic-like experiences in clinical and non-clinical adolescents that revealed a clear association between “psychotic” (e.g., paranoia, hallucinations) and emotional disturbances, which challenged the historical divide between them [20, 21, 22]. From another point of view, Kruger’s paper [6] argued PPS was linked to insecurity in interpersonal relationships, a convincing argument that was indirectly supported, from another point of view, by our data. The observed link between PPS and emotional problems may be due to personality factors not explored in the present papers, but highlighted into a previous one [11]; for example, it is known that conscientiousness is negatively related to frequency of PPS and neuroticism is positively related to bothersomeness of PPS; future studies may systematically analyze a model with personality factors *and* metal health problems in relationship to PPS. Back to the previous results on gender, it makes sense that as females are more prone to experience internalizing symptoms [23], they are more prone to display PPS as well.

Regarding other results, even if we used smartphone-related variables (phone use and phone check, which were self-reported) as control variables, we confirmed that both are factors that explain part of the individual differences in the experience of PPS, as reported by other authors [1, 3]. More doubtful is the relationship with conduct problems, as our data were basically exploratory and need replication. The alpha value of the SDQ conduct problem subscale was poor and thus it may not be the best way to assess non-clinical conduct problems. Anyway, temper tantrums seem associated to PPS in both models, thus it seems useful to explore the relationship between externalizing symptoms and PPS in future studies. Reward processing-related circuits, which are activated by social media use [24], are activated by aggression as well [25] and thus a link may be postulated and justified, but specific data on PPS are lacking.

The theoretical implications of our findings are related to the conceptualization of PPS. Considering PPS a hallucination (auditory or tactile) that is not schizophrenic in nature, but rooted in a background that contains emotional, stress-related [4, 5] and attachment/interpersonal [6] difficulties seems the best way to conceptualize the phenomenon. From a clinical point of view, it seems important to detect PPS when present as it may represent an alarm for possible emotional problems, but it is far from being a signal of severe mental illness. Whether it is a precursor/risk factor or a simple correlate needs to be determined.

The results should be interpreted in light of some limitations. First, all data are self-reported which may be a source of bias (for example, a social desirability effect, which may cause a subject to answer positively to several questions, could have inflated the observed correlation). While it may be suitable to assess emotional problems from teachers’ or parent’s reports allowing future studies can overcome this limitation, it seems difficult to find a different way to assess PPS; in this regard, our intensity question referred to “how often do subjects feel the phenomenon” without assessing the bother or impact on everyday life, which may have limited the study. A more comprehensive measure of PPS would be welcome in the future. Also, we had not measured any stress-related variables or socio-economic assessment; thus, the results may be, at least in part, driven by these other variables. Finally, alphas of SDQ-subscales resulted to be poor to moderate. Replication with more reliable instruments is needed.

In conclusion, the established link of PPS with emotional psychopathology opens the routes for future studies that could examine the phenomenon in clinical samples or in relation to more specific dimensions of psychopathology (e.g., externalizing symptoms, obsessive-impulsive spectrum). More importantly, future studies could address these issues longitudinally. For now, we advise mental health professionals, as well as caregivers, that PPS is prevalent in youths, and may be a signal of emotional problems.

## Supporting information

S1 File (XLS)

S1 Table (ODT)
