# Supplementary material for: Phantom Phone Signals in youths: Prevalence, correlates and relation to psychopathology
Source: PLoS One. 2019 Jan 4;14(1):e0210095. doi: 10.1371/journal.pone.0210095 (PMC6319810; doi:10.1371/journal.pone.0210095)
Supplement: Supplementary file 1 [file pone.0210095.s002.odt]

Table 3 Multivariate regression model with the dependent variable PPS (Likert scale), N=2859

| PPS (n. 2859) |  |  |
| --- | --- | --- |
|  | B (95% CI) | p-value |
| Age | 0.18 (0.06-0.29) | 0.02 |
| Gender (Male/Female) | 0.02 (-0.2-0.2) | 0.8 |
| Smarttime | 0.22 (0.18-0.26) | 0.000 |
| Smartcheck | 0.3 (0.27-0.33) | 0.000 |
| SDQ-EP | 0.10 (0.05-1.6) | 0.000 |
| SDQ-HY | 0.04 (-0.01-1) | 0.13 |
| SDQ-PP | 0.029 (-0.03-0.9) | 0.39 |
| SDQ-PSB | 0.03 (-0.03-0.9) | 0.34 |
| Tantrum | 0.2 (0.02-0.38) | 0.02 |
| Obey | -0.5 (-0.24-1.3) | 0.57 |
| Fight | 0.9 (-0.1-0.3) | 0.46 |
| Lie | -0.02 (-0.2-1.6) | 0.83 |
| Steal | 0.06 (-0.1-0.3) | 0.64 |
| R2 | 0.26 |

Legend: PPS: Phantom Phone Signal; SDQ-EP: SDQ Emotional Problems; SDQ-CP: SDQ Conduct Problems; SDQ-HY: SDQ Hyperactivity; SDQ-PP: SDQ Peer Problems; SDQ-PSB: SDQ Pro-social Behaviour.
